# Adenocarcinoma in Intraductal Tubulopapillary Neoplasm of the Pancreas: A Case Report and Literature Review

**DOI:** 10.7759/cureus.43006

**Published:** 2023-08-05

**Authors:** Komson Wannasai, Chanakrit Boonplod, Tarathep Wongsuriyathai, Amonlaya Amantakul, Sunhawit Junrungsee, Sarawut Kongkarnka

**Affiliations:** 1 Department of Pathology, Faculty of Medicine, Chiang Mai University, Chiang Mai, THA; 2 Department of Radiology, Faculty of Medicine, Chiang Mai University, Chiang Mai, THA; 3 Department of Surgery, Faculty of Medicine, Chiang Mai University, Chiang Mai, THA

**Keywords:** intraductal tubulopapillary neoplasm, pancreas disease, case report, adenocarcinoma, intraductal tubulopapillary neoplasm of the pancreas

## Abstract

Intraductal tubulopapillary neoplasms (ITPNs) are a subgroup of pre-malignant pancreatic epithelial lesions. The histomorphological and immunophenotypical characteristics of ITPN have been described by several authors based on case series; however, the rarity of this tumor subtype and its similarity to other entities makes the identification of ITPN challenging for radiologists and pathologists. Herein, we report a case of ITPN with associated invasive carcinoma along with a literature review that will benefit further studies and help in planning treatments for patients in the future. A pancreatic mass was incidentally discovered in a 40-year-old woman during her annual check-up. Radiological investigation revealed a mass that obstructed the main pancreatic duct and caused ductal dilatation. Endoscopic retrograde cholangiopancreatography with biopsy indicated poorly differentiated adenocarcinoma. Subsequently, total pancreatectomy with splenectomy was performed to remove the tumor. ITPN of the pancreas with associated poorly differentiated adenocarcinoma was diagnosed based on pathological and immunohistological test results. Achieving complete resection of the tumor, the patient did not require chemotherapy during follow-up care. Thus, our study demonstrated the necessity of radiological and histopathological correlation in the definitive diagnosis of pancreatic ITPN. However, the determination of an invasive component is essential because malignant transformation affects the prognosis of patients.

## Introduction

Tajri et al. first described pancreatic intraductal tubulopapillary neoplasm (ITPN), also known as intraductal tubular carcinoma in 2004 [1]. In 2009, Yamaguchi et al. established the term “intraductal tubulopapillary neoplasm" [2]. In 2010, the World Health Organization (WHO) acknowledged ITPNs as a separate entity within a subgroup of pre-malignant epithelial pancreatic tumors [3]. It was classified as an intraductal, grossly apparent tubule-forming epithelial tumor with high-grade dysplasia and ductal differentiation but no apparent mucin production. ITPN is a rare neoplasm that accounts for less than 1% of all exocrine pancreatic tumors [4]. It often manifests as a solid mass occupying the major pancreatic duct, resulting in duct dilation [5].

The clinical manifestations and imprecise radiographic findings of ITPN may make diagnosis challenging [6]. Various publications have documented the histomorphological and immunophenotypical characteristics of ITPN based on the case series. However, the rarity of this tumor subtype and its similarities to other entities make diagnosing ITPN difficult for radiologists and pathologists.

Although the histopathological and immunomorphological characteristics of ITPN have been extensively studied, there is limited information on the surgical outcome and prognosis of ITPN. Herein, we presented a case of ITPN associated with invasive carcinoma in a patient from our hospital for educational purposes, including the diagnosis and management of other patients.

## Case presentation

Patient history

A 40-year-old woman with underlying ductal carcinoma in situ of her right breast visited a local health center for an annual health screening after breast mass excision. During her surveillance abdominal ultrasound, a pancreatic mass was incidentally discovered. She was referred to our hospital, where an endoscopic ultrasound-guided biopsy was performed. Preoperative laboratory examination revealed no anemia with leukocytosis (neutrophil predominance), no coagulopathy, normal serum electrolyte levels, and normal liver function test results. Alpha-fetoprotein and cancer antigen (CA) 19-9 levels were negligible.

Imaging study

**Figure 1:**
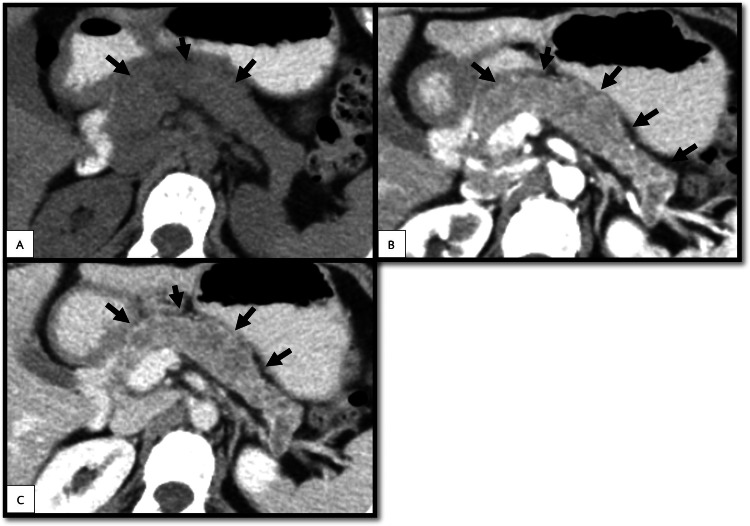
Abdominal computed tomography (CT) scan (arrow, the tumor) Unenhanced CT scan (A) revealed an isoattenuating to slightly low-attenuating tumor. Contrast-enhanced CT (B, arterial phase; C, portovenous phase) revealed the hypovascular tumor inside the dilated main pancreatic duct.

Endoscopic ultrasonography revealed a diffuse hypoechoic and enlarged edematous pancreas with a peripancreatic capsule. The patient’s abdominal computed tomography (CT) (Figure 1) revealed a mass within the dilated main pancreatic duct from head to tail. Non-contrast scanning revealed an isoattenuating to slightly low-attenuating mass without fat or calcification. Dynamic contrast imaging revealed hypoenhancement compared with the pancreatic parenchyma. No evidence of chest or abdominal metastases was detected. These findings were inconclusive, and the tumor type was uncertain.

**Figure 2:**
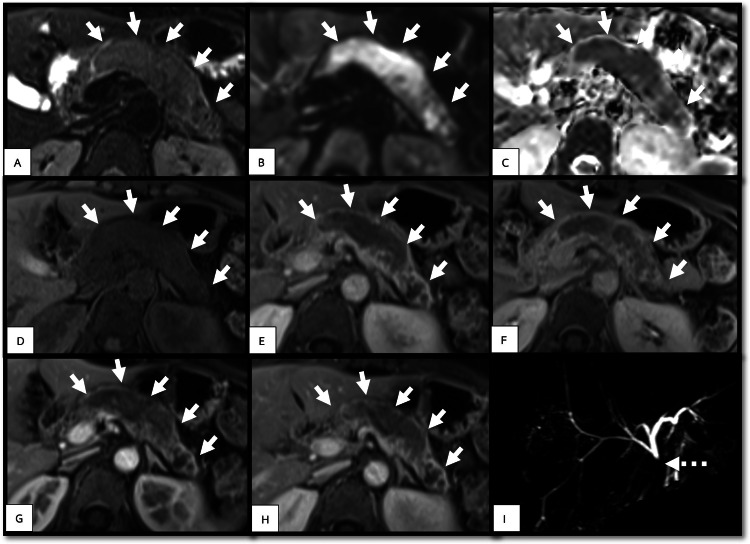
Abdominal MRI and MRCP (solid arrow, tumor; dotted arrow, common bile duct) The MRI (A, FS-T2WI; B, DWI; C, ADC; D, unenhanced FS-T1WI; E–H, dynamic contrast-enhanced FS-T1WI in sequential order; I, MRCP) revealed the mass to be isointense to slightly hypointense on FS-T1WI, slightly hyperintense on FS-T2WI, restricted diffusion on DWI, and hypointense on ADC. After contrast injection, the mass appeared hypovascular compared with the pancreatic parenchyma. The common bile duct in the area of the tumor was abruptly disrupted, according to the MRCP. (ADC, apparent diffusion coefficient; DWI, diffusion-weighted imaging; FS, fat suppression; MRCP, magnetic resonance cholangiopancreatography; MRI, magnetic resonance imaging; WI, weighted imaging).

Magnetic resonance imaging (MRI) and magnetic resonance cholangiopancreatography (MRCP) were performed to obtain additional information. MRI and MRCP (Figure 2) revealed the same findings as CT. The mass was hypovascular within the dilated main pancreatic duct. Additionally, MRCP revealed that the mass obstructed the distal common bile duct.

Based on the imaging findings, the mass was likely within the main pancreatic duct, causing obstruction and dilatation of the duct. No vascular invasion, lymphadenopathy, or distant metastases were observed, and the tumor was likely resectable.

Patient management and pathologic findings

**Figure 3:**
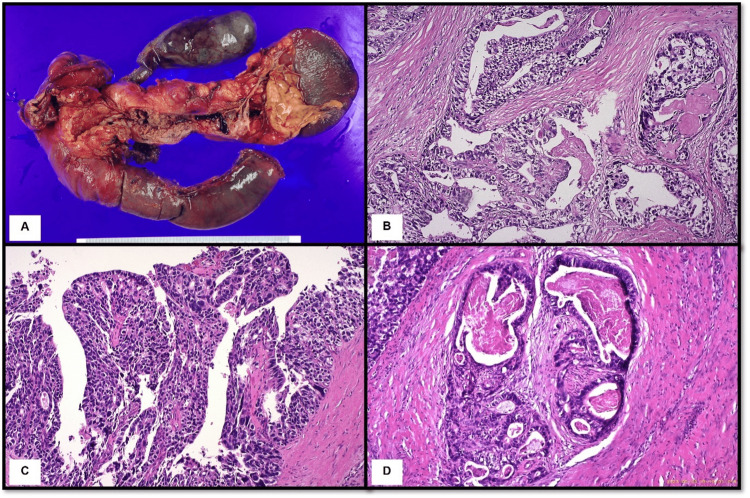
Gross and microscopic pathology of the total pancreatectomy with splenectomy specimen (A) A longitudinal opening of the main pancreatic duct revealed an intraductal growth of papillary-forming tumor; (B) Tumor section revealing tubular structure (hematoxylin and eosin, H&E; 200×) lined with large, atypical cells with pleomorphic hyperchromatic nuclei, prominent nucleoli, and a moderate amount of cytoplasm; (C) Tumor section revealing tumor cells arranged in a papillary structure (H&E, 200×); (D) Tumor section revealing perineural invasion (H&E, 200×).

The patient underwent endoscopic retrograde cholangiopancreatography (ERCP) for pancreatic biopsy. The specimen obtained via endoscopy was subjected to histopathological and immunohistochemical (IHC) analyses, which revealed the presence of a partially necrotic, poorly differentiated pancreatic tumor that was positive for cytokeratin (CK) 7 and negative for the cluster of differentiation (CD) 56, synaptophysin, chromogranin A, caudal-type homeobox 2 (CDX2), and CK20. The proliferation rate determined by Ki-67 staining was 60%. The provisional diagnosis was poorly differentiated pancreatic adenocarcinoma, and the absence of immunohistological reaction to all neuroendocrine markers excluded a neuroendocrine tumor diagnosis. Subsequently, a total pancreatectomy with splenectomy was performed to remove the tumor. Macroscopic pathology (Figure 3) revealed intraductal growth of a friable papillary-like mass in the main pancreatic duct. The mass was 8 cm long, with a maximum diameter of 2.5 cm. The spleen, duodenum, and gallbladder were also unremarkable. Histological examination (Figure 3) of the hematoxylin and eosin-stained intraductal mass sections revealed atypical cells predominately in tubular growth patterns with back-to-back tubular glands and solid sheets with focal papillary formations. With the perineural invasion, the tumor cells spread beyond the main pancreatic duct with the size of invasive foci in approximately 1.2 cm. All surgical margins were tumor-free except for the common bile duct margin, which was positive for an intraductal papillary neoplasm of the bile duct.

**Figure 4:**
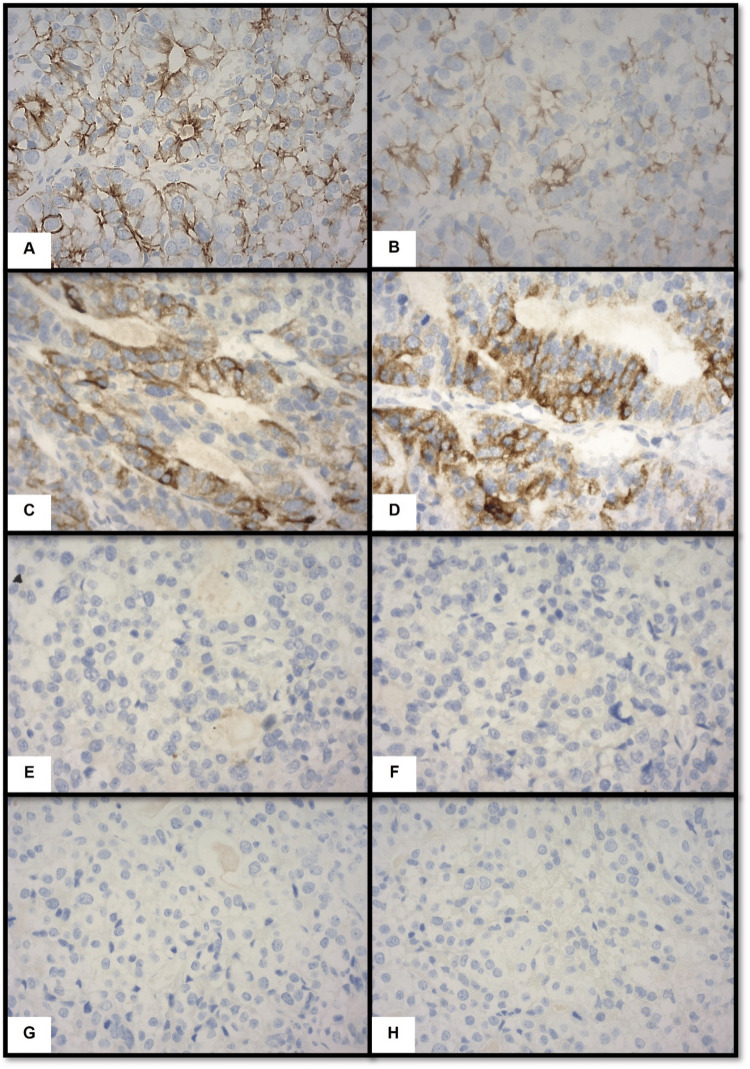
Immunohistochemical staining of the tumor (400×) (A) CK7, (B) CK19, (C) MUC1, (D) MUC6, (E) MUC2, (F) MUC5AC, (G) hepatocyte antigen, and (H) CD56. (CK, cytokeratin; MUC, mucin; CD, cluster of differentiation)

IHC staining (Figure 4) revealed that the tumor cells were positive for CK7, CK19, mucin (MUC) 1, MUC6, CA19-9, carcinoembryonic antigen, E-cadherin, and p16 but negative for CD56, synaptophysin, chromogranin A, MUC2, MUC5AC, CA12-5, hepatocyte antigen, and p53. All dissected lymph nodes were negative for tumors. Based on histomorphological and IHC staining, a diagnosis of ITPN of the pancreas with associated poorly differentiated adenocarcinoma was made.

Clinical course

No complications were observed following the surgical removal of the pancreatic tumor. No chemotherapy was prescribed due to the complete resection of the tumor. Three months after surgery, MRI upper abdomen revealed two new enhancing liver nodules in hepatic segments 6 and 7, and positron imaging (PET/CT) was suggestive of liver metastases without local tumor recurrence. A liver biopsy was performed, and metastatic breast adenocarcinoma was considered histologically and immunohistochemically. The patient will be given chemotherapy for the treatment of metastatic breast cancer.

## Discussion

ITPN is a rare intraductal pancreatic epithelial neoplasm. The disease was initially reported by Yamaguchi et al. in 10 patients, where they detailed the ITPN features discovered in those patients [2]. In 2019, the 5th edition of the WHO tumor classification identified ITPN as a pancreatic intraductal neoplasm [3].

Common symptoms of ITPN, such as stomach discomfort and bloating, are nonspecific; therefore, patients rarely seek medical advice during the initial stages of the disease [7]. Most lesions are accidentally discovered through ultrasonography or other radiographic examinations [7]. Similarly, the tumor was discovered accidentally during annual abdominal ultrasounds in this case. However, obtaining information from radiographs is challenging because the condition radiographically resembles other diseases, such as intraductal papillary mucinous neoplasm pancreas (IPMN) and pancreatic ductal adenocarcinoma (PDAC) [8].

Owing to the limited number of publications describing the imaging characteristics of ITPN, it is challenging for radiologists to diagnose ITPN before surgery accurately. A review of previous case reports revealed that ITPN could develop in any part of the pancreatic duct; some cases are focal, whereas others are diffuse [6, 9-12]. Five case reports revealed a diffuse pattern with tumors located in the head and neck [9], head and body [10], or body and tail of the pancreas [11, 12]. No previous report of a tumor that extends from the head to the tail exists, as in this case. According to a literature review by Zhang et al., ITPN revealed a solid component in approximately 78% of cases, which was discovered in 28 of 36 cases; CT and MRI of ITPN revealed a hypovascular mass with hypointense T1-weighted images, slightly hyperintense T2-weighted images, and hyperintense diffusion-weighted imaging [6]. However, based on imaging findings, ITPN should be differentiated from IPMNs, which are the most common intraductal tumors. IPMNs are typically cystic, whereas ITPNs are typically solid.

ITPN often manifests as a solid, nodular tumor that emerges from the primary pancreatic duct and seldom affects branch ducts [2]. The tumor cells exhibit high-grade dysplasia and lack intracellular mucin. However, the cellular features are comparable to those of pancreatic adenocarcinoma and neuroendocrine tumor [13]. IHC staining can assist in diagnosis [14]. 

ITPN is positive for the pancreatic duct differentiation markers CK7, CK19, MUC1, and MUC6 [7, 8, 15] but negative for CDX2, MUC2, and MUC5AC [7]. These negative IHC results demonstrated no gastroenteric differentiation [8]. Muraki et al. discovered that ITPN could be distinguished from IPMN by MUC5AC staining; ITPN is negative, and IPMN is positive for MUC5AC staining [16]. Moreover, ITPN in the main pancreatic duct is positive for CK7 and CK19 but negative for MUC2 and MUC5AC, as reported by Fujimoto et al. [17].

In our case, it was difficult to establish a definitive diagnosis from a biopsy sample due to the small size of the samples and the presence of tumor cells with atypia. The tumor cells were positive for CK7 and CK19 upon IHC labeling but negative for CD56, chromogranin A, and synaptophysin. A tissue biopsy led to a diagnosis of poorly differentiated pancreatic adenocarcinoma. After acquiring all pancreatectomy samples, we discovered that most lesions began from the main pancreatic duct and subsequently migrated to the neighboring minor duct, demonstrating tumor cell growth throughout the pancreas. The tumor cells possessed the same immunophenotype as the biopsy samples. Therefore, we concluded that the patient had pancreatic ITPN.

Basturk et al. and Paolino et al. discovered that approximately 70% and 60% of ITPN cases were associated with invasive carcinoma, respectively [7, 15]. Invasive foci have features comparable to PDAC; biopsy of the tumor cells, including invasive cells, may lead to a false diagnosis of PDAC. Typically, invasive foci of ITPN are discovered in areas where the tumor boundary is irregular and occasionally in areas that resemble intraductal growth [13]. In our case, the main feature of the invasive foci was the presence of tiny irregular glands penetrating the pancreatic tissue. Perineural invasion was also observed, confirming that ITPN was associated with invasive carcinoma.

ITPN is genetically different from IPMN and PDAC. ITPN is distinguished from IPMN and ductal adenocarcinoma primarily by the absence of KRAS and BRAF mutations [2, 7, 8, 18, 19]. In addition, the usual pancreatic regulators (CDKN2, TP53, GNAS, SMAD4, and RNF43) were less changed in ITPN compared to PDAC [15]. Unfortunately, in our case, mutation detection was performed for only the breast cancer gene BRCA1, which was negative. Based on the patient’s medical history, ductal carcinoma in situ of the breast without a BRCA1 mutation was identified.

Several studies have indicated that ITPN has a favorable prognosis and can be resected more effectively than PDAC [18, 20, 21]. For example, Basturk et al. studied 22 patients and discovered that ITPN without aggressive cancer had a 100% 5-year survival rate [7]. Patients with ITPN-associated invasive carcinoma had a 71% likelihood of survival within 5 years. In a study by Kolby et al., a 42-year-old man with upper abdominal pain was diagnosed with multifocal ITPN and invasive growth [19]. A comprehensive pancreatectomy was performed. The patient received 6 months of adjuvant chemotherapy with gemcitabine-capecitabine, and the postoperative course was uneventful. The patient was alive 19 months after the procedure without any indications of recurrence. Paolino et al. reported a type of ductal tree involvement that is considered a potentially crucial prognostic factor [15]. Mixed-duct involvement correlated with a higher risk of ITPN recurrence as compared to involvement of the main duct and branch ducts; the branch duct involvement showed the lowest risk of recurrence. In the postoperative period of 3 months following oncologic R0 resection, our patient exhibited no signs of local recurrence of the pancreatic tumor; however, hepatic metastases due to breast cancer were discovered. The patient will be administered chemotherapy and closely followed up for metastatic disease.

## Conclusions

ITPN are rare pancreatic diseases that require histological and immunochemical examinations to establish a definitive diagnosis. It is important to highlight that this tumor can be misdiagnosed as PDAC based on a biopsy alone. Therefore, accurate diagnosis hinges on comprehensive clinico-radio-pathological correlations. Obtaining a preoperative diagnosis poses significant challenges. It is noteworthy that the majority of intraductal pancreatic tumors are diagnosed through ERCP and subsequent biopsy. However, relying solely on this method can potentially lead to frequent misdiagnosis of ITPN. Given the propensity for invasiveness, it is crucial to conduct thorough examinations and obtain comprehensive tumor samples, particularly when compared to other intraductal neoplasms. While most cases of ITPN are benign, a subset displays features of invasive carcinoma, such as local invasion and perineural invasion, as demonstrated in our case.
